# Lentiviral delivery of combinatorial CAR/CRISPRi circuit into human primary T cells is enhanced by TBK1/IKKɛ complex inhibitor BX795

**DOI:** 10.1186/s12967-020-02526-2

**Published:** 2020-09-23

**Authors:** Lingyu Li, Yuan Gao, Richa Srivastava, Wei Wang, Qinghui Xiong, Zhiming Fang, Alejandra Pelayo, Carolyn Denson, Angshumala Goswami, Rona Harari-Steinfeld, Zhifen Yang, Lihong Weng, Lei Stanley Qi, Francesco M. Marincola

**Affiliations:** 1 Refuge Biotechnologies Inc., Menlo Park, CA 94025 USA; 2 Hangzhou Juwu Biotech Co., Ltd., Hangzhou, 310018 Zhejiang China; 3 Department of Bioengineering, Department of Chemical and Systems Biology, ChEM-H, Stanford University, Stanford, CA USA 94305

**Keywords:** BX795, Lentiviral transduction, T cell engineering, CAR-T, CRISPRi

## Abstract

**Background:**

Adoptive transfer of engineered immune cells is a promising strategy for cancer treatment. However, low transduction efficiency particularly when large payload lentiviral vectors are used on primary T cells is a limitation for the development of cell therapy platforms that include multiple constructs bearing long DNA sequences. RB-340-1 is a new CAR T cell that combines two strategies in one product through a CRISPR interference (CRISPRi) circuit. Because multiple regulatory components are included in the circuit, RB-340-1 production needs delivery of two lentiviral vectors into human primary T cells, both containing long DNA sequences. To improve lentiviral transduction efficiency, we looked for inhibitors of receptors involved in antiviral response. BX795 is a pharmacological inhibitor of the TBK1/IKKɛ complex, which has been reported to augment lentiviral transduction of human NK cells and some cell lines, but it has not been tested with human primary T cells. The purpose of this study was to test if BX795 treatment promotes large payload RB-340-1 lentiviral transduction of human primary T cells.

**Methods:**

To make the detection of gene delivery more convenient, we constructed another set of RB-340-1 constructs containing fluorescent labels named RB-340-1F. We incorporated BX795 treatment into the human primary T cell transduction procedure that was optimized for RB-340-1F. We tested BX795 with T cells collected from multiple donors, and detected the effect of BX795 on T cell transduction, phenotype, cell growth and cell function.

**Results:**

We found that BX795 promotes RB-340-1F lentiviral transduction of human primary T cells, without dramatic change in cell growth and T cell functions. Meanwhile, BX795 treatment increased CD8+ T cell ratios in transduced T cells.

**Conclusions:**

These results indicate that BX795 treatment is effective, and might be a safe approach to promote RB-340-1F lentiviral transduction of human primary T cells. This approach might also be helpful for other T cell therapy products that need delivery of complicated platform via large payload lentiviral vectors.

## Background

T cells engineered with chimeric antigen receptors (CARs) represent a promising strategy for cancer treatment. However, many challenges remain in eradicating tumors [1, 2], including antigen escape [3, 4], immunosuppression in the tumor microenvironment [1, 5, 6] and lack of strictly tumor-specific targets [7]. To overcome these hurdles, incorporation of multiple strategies which can enhance antitumor efficacy and improve safety are required, and increasingly more combination strategies and synthetic biological approaches emerged. For instance, pre-clinical and clinical studies demonstrated that combining CAR-T with PD-1 blockade, either through targeted delivery of anti-PD-1 scFvc or genetic knock out of the PD-1 gene, shows enhanced tumor control compared with conventional CAR-T [8–11]. Synthetic regulatory circuits, like SynNotch/CAR, have been constructed to enable T cell activation to broaden the tool box for the design of more specific and safer T cell therapies for a wider range of tumor types [12].

Here, we present RB-340-1 as a combinatorial CAR/CRISPRi circuit that couples CRISPR interference (CRISPRi) to CAR signaling upstream and downstream to gene suppression of the endogenous Pd-1 gene to prevent CAR T cell exhaustion and enhance clinical outcomes. CRISPRi is a non-editing gene expression regulation strategy based on the nuclease de-activated CRISPR associated (dCas9) protein [13–15]. Coupling of dCas9 to a transcriptional repressor domain could silence expression of multiple endogenous genes [14]. RB-340-1 is engineered to express an anti-HER2 CAR single chain variable fragment (scFv) (4D5 clone) [16], with CD28 and CD3ζ co-stimulatory domains linked to a tobacco etch virus (TEV) protease and a PD-1 promoter region-targeting short guide RNA (PD1sg). The other complex of RB-340-1 includes linker for activation of T cells (LAT) complexed to dCas9-Kruppel-associated box (Krab) via a TEV-cleavable linker. Upon antigen- encounter, the LAT-dCas9-Krab complex is cleaved by the TEV allowing nuclear translocation of dCas9-Krab to the transcription start site of the PD-1 gene. This conditional, non-gene editing and reversible suppression promotes resilience to checkpoint inhibition, and in vivo persistence and effectiveness against HER2-expressing oropharyngeal cancer xenografts. To make the detection of gene delivery more convenient, we constructed another set of RB-340-1 constructs containing fluorescent labels, which is named as RB-340-1F.

Currently, lentiviral transduction is a favorite method applied to deliver genes into T cells [17–21]. However, the efficiency of transduction of primary T cells with large payload lentiviral vectors is very low, especially when multiple lentiviral vectors are needed for transduction. To overcome this hurdle, we tried to utilize inhibitors of receptors involved in antiviral response. It is very well studied that TANK-binding kinase 1 (TBK1) and IƙB kinase ɛ (IKKɛ) regulate the production of type 1 interferons (IFNs), which trigger antiviral responses during viral infections [22, 23]. Therefore, blocking TBK1 activity should prevent the induction of interferons, reduce host response, and facilitate viral infection [24–26]. The compound BX795 has been reported as an inhibitor of the TBK1 and IKKɛ complex [27]. Moreover, a study in human natural killer (NK) cells found that BX795 boosted on average 3.8-fold the efficiency of lentiviral genetic modification [28]. Significant promotion of transduction was seen at 2 µM–10 µM concentration, without causing toxic effects on NK cells. Transduction efficiency only increased when BX795 was present during the transduction and did not increase when NK cells were pretreated and BX795 was absent during transduction, which means that the inhibition of antiviral response by BX795 is reversible. BX795 was also shown to promote lentiviral transductions in different cell lines. However, BX795 treatment has not been tested with human primary T cells yet. In this study, we use BX795 to promote RB-340-1F lentiviral transduction of human primary T cells and also detected the changes that BX795 treatment might bring to the transduced T cell product.

## Methods

### Cell lines

293T cells were purchased from ATCC (Manassas, VA) and maintained in Dulbecco’s modified Eagle’s medium (DMEM, Invitrogen) supplemented with 10% Fetal Bovine Serum (FBS, Corning), and 1% Penicillin–Streptomycin (Invitrogen). The human head and neck squamous cell carcinoma line FaDu was obtained from the ATCC (Manassas, VA). FaDu/PD-L1 was generated by transducing lentiviral vector, pLenti6.3/V5TM-TOPOTM (Invitrogen), overexpressing PD-L1 (Human cDNA, NM_014143.4), followed by blasticidin selection. Fadu-PDL1 cells were maintained in the same medium as 293T cells.

### Lentiviral vectors production and titration

Second-generation, self-inactivating lentiviral vectors were produced with 293T cell line. 3 × 10^7^ 293T cells are seeded in T175 cell culture flasks. After 24 h, 70%–80% confluent 293T cells were transfected with 108 µl TransIT-LT1 (Mirus) mixed with 32 µg lentiviral vector plasmid, 16 µg psPAX2, and 8 µg pMD2.G packaging plasmid. ViralBoost Reagent (ALSTEM) was added to facilitate virus production. Viral supernatant was harvested 72 h post transfection. After clarification, the supernatant was concentrated by centrifugation at 10,000*g* through a 10% sucrose cushion for 4 h at 4 °C. The pelleted vectors were resuspended in RPMI 1640 Media (Gibco), aliquoted, and stored at − 80 °C for later use.

Lentiviral vector was titrated on HT1080 cells by serial dilution in DMEM supplemented with 10% FBS. 72 h post infection, the ratio of GFP or mCherry positive cells was detected by flow cytometry. Titer-transducing units/mL (TU/mL) was calculated using the following formula: Titer (TU/mL) = (N × P)/(V × D), where N = number cells per well, P = percent fluorescent positive cells, V = volume (μL) virus per well, D = fold dilution.

### Human primary T cell isolation

Healthy donor leukopaks were purchased from PPA Research Group (pparesearch.com) and Miltenyi Biotec. Fresh peripheral blood mononuclear cells (PBMCs) were isolated by low-density centrifugation on Lymphoprep (Stem Cell Technology) according to the manufacturer’s instructions. CD3+ T cells were purified by negative selection using the Pan T Cell Isolation Kit Dynabeads Untouched Human T Cells (Invitrogen). In some experiments, T cells were enriched by CD4/CD8 positive selection using the StraightFrom^®^ Leukopak^®^ CD4/CD8 MicroBead kit, human (Miltenyi Biotec) and MultiMACS™ Cell24 Separator Plus (Miltenyi Biotec) according to the manufacturer’s instructions.

### Lentiviral transduction of T cells

On day 0, cryopreserved Pan T cells or CD4+/CD8+ T cells were thawed and activated in 24-well plates pre-coated with anti-human CD3 antibody (Clone OKT3, 1 µg/ml, BioLegend) and anti-human CD28 antibody (Clone CD28.2, 1 µg/ml, BioLegend). T cell culture medium was composed of RPMI 1640 (Gibco), 10% Human Serum AB (GeminiBio), 2 mM GlutaMAX (Gibco), 50 µM 2-Mercaptoethanol (Gibco), 100 U/ml penicillin and 100 µg/ml streptomycin (Gibco), 10 ng/ml recombinant human IL-7 (Gibco) and 10 ng/ml IL-15 (Gibco). On day 1, LdCK-GFP lentiviral vector and transduction enhancer TransPlus (Alstem) was added to T cell culture (total volume: 0.5 ml/well) with or without BX795 (final concentration 4 µM). Six hours after transduction, 1 ml fresh medium was supplemented to each well to support cell growth for overnight. On day 2, 1 ml supernatant was removed from each well. CAR-TEV-mCherry lentiviral vector and transduction enhancer TransPlus (Alstem) was added to T cell culture (total volume: 0.5 ml/well) with or without BX795 (final concentration 4 µM). Six hours after transduction, 1 ml fresh medium was supplemented to each well. On day 5, medium was changed, lentiviral vectors and all supplementary were washed out. Meanwhile, T cell stimulation was withdrawn via transferring cells into new plates. On day 6, GFP + and mCherry + double-transduced T cells were enriched through cell sorting (Sony SH800 Cell Sorter). The enriched cells were expanded until day 14 or day 21.

### T cell killing assay

The cytotoxic capacities of the final products were evaluated in vitro against Fadu/PD-L1 tumor cells. In short, a total of 8 × 10^4^ Fadu/PD-L1 target cells in 400 µL medium (DMEM supplemented with 10% FBS and 1% Penicillin–Streptomycin) was placed in triplicates into 48-well plates and incubated overnight for attachment. The following day, 400 µL of effector cells were added to obtain effector-to-target (E:T) ratios at 1:5 or 1:20. The amount of effector cells was calculated as CAR positive cells. 24 h after co-culture, supernatants were harvested to measure the secretion of IL-2, TNFα, and IFN-ɣ by following the directions of ELISA kits (DuoSet ELISA Development System, R&D Systems). Cells were collected for flow cytometry assay in the following 3 days.

### Flow cytometry

Cells were washed once with FACS buffer composed of 1x DPBS without Ca^2+^ and Mg^2+^ (Invitrogen) and 2% heat-inactivated FBS(Corning), then incubated with appropriate amounts of antibody at 4 °C for 30 min, protected from light. The labeled cells were then washed and re-suspended in FACS buffer. For cell-mediated cytotoxicity assay, CountBright™ absolute counting beads (Invitrogen) were added and mixed with cells prior to data acquisition. Data acquisition was performed on CytoFLEX flow cytometer (Beckman Coulter). Data were analyzed through Kaluza software (Beckman Coulter). LdCK-GFP and CAR-TEV-mCherry expression was determined by fluorescent labels. In some experiment, LdCK and MSLN-CAR-TEV without fluorescent labels were used, their expression was assessed through detecting their tags with antibodies Q8-PE (clone QBEND/10, ThermoFisher) and NGFR-FITC (clone ME20.4, BioLegend). The antibodies used for T cell phenotyping were CD3-APC (clone SK7), hCD45-AF700 (clone HI30), CD4-PerCP (clone SK3), CD8-BV510 (clone SK1), CD27-PE-Cy7 (clone M-T271), CD28-BV605 (clone CD28.2), and CD279/PD1-BV421 (clone EH12.2H7) from BioLegend. The antibodies used for cell-mediated cytotoxicity assay were CD274/PDL1-PE-Cy7 (clone MIH1, Invitrogen), CD340/HER2-BV605 (clone 24D2, BD Biosciences), CD3-APC (clone SK7), hCD45-AF700 (clone HI30), CD4-PerCP (clone SK3), CD8-BV510 (clone SK1), and CD279/PD1-BV421 (clone EH12.2H7) from BioLegend. Live/dead discrimination was determined using LIVE/DEAD^®^ Fixable Near-IR Dead Cell Stain (Invitrogen).

### Statistical analysis

For preparation of graphs and statistical analysis, Microsoft Excel and GraphPad Prism8 were used. P < 0.05 was considered statistically significant. Significance of findings was defined as: ns = not significant; *, P < 0.05; **, P < 0.01.

## Results

### Establishment of human primary T cell transduction procedure for RB-340-1F

**Fig. 1:**
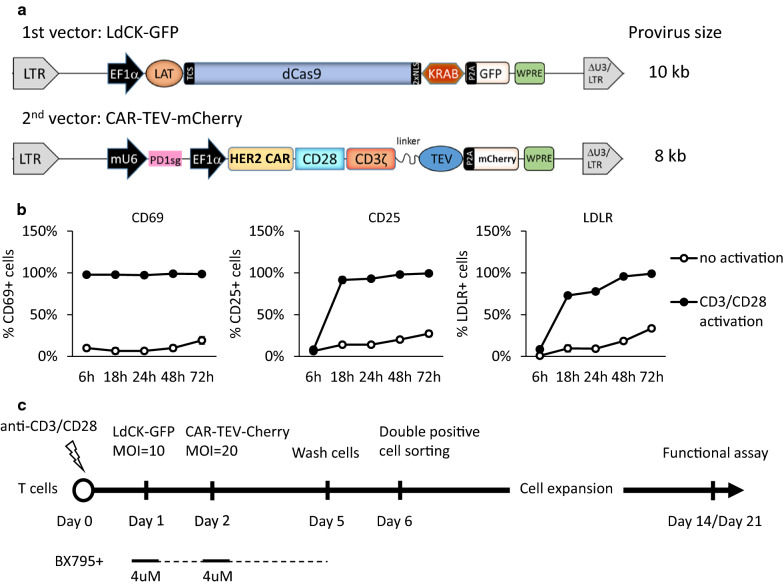
RB-340-1F two-vector platform and T cell transduction workflow. **a** Schematic representation of the first lentiviral vector LdCK-GFP and the second lentiviral vector CAR-TEV-mCherry. **b** The percentages of cells expressing the activation markers CD69, CD25, and cell surface protein LDLR. T cells were stimulated in anti-CD3/anti-CD28 pre-coated plate (CD3/CD28 activation). T cells cultured in non-coated plate were used as control (no activation). Results are the averages of two donors. **c** T cell transduction workflow and the time window to add BX795. The final concentration of BX795 in cell culture is 4 µM

RB-340-1 is a combinatorial CAR/CRISPRi circuit delivered into T cells by two lentiviral constructs, LdCK and CAR-TEV (Fig. 1a). LdCK encodes linker for activation of T cells (LAT) complexed to dCas9-Krab via a TEV-cleavable sequence (TCS). CAR-TEV encodes an anti-HER2 (4D5) CAR single chain variable fragment (scFv), with CD28 and CD3ζ co-stimulatory domains linked to a tobacco etch virus (TEV) protease and a short guide RNA (PD1sg) targeting the transcription start site (TSS) of the PD-1 gene. The integrated provirus sequence between two LTRs is around 10 kb for LdCK, and is around 8 kb for CAR-TEV. In this study, green fluorescent protein and m-cherry were used as reporters in LdCK and CAR-TEV, respectively. To distinguish the platform using fluorescent labels from RB-340-1 without fluorescent labels, we name it as RB-340-1F.

Human primary T cells need to be activated before transduction [19, 29–31]. To identify the proper timing for transduction, we documented the status of activation of primary T cells by testing the level of expression of canonical activation markers following stimulation with anti-CD3 plus anti-CD28 (Fig. 1b). CD69 is an early T cell activation marker [32] and was detectable within hours of stimulation. CD25 is the alpha chain of the IL-2 receptor [33] and became up-regulated 18 h after stimulation. Low-density lipoprotein-receptor (LDLR), the major entry port of VSV-G pseudo-typed lentiviral vectors in human cells [34] was also up-regulated 18 h after stimulation. Thus, we selected 18 h as the earliest time point to expose lentivirus to T cells. Lentiviral transduction was tried at different time windows from 18 h to 72 h. Several other factors that affect lentiviral transduction, including virus multiplicity of infection (MOI) and application of transduction enhancers like Transplus, were also investigated. Based on these preliminary tests, we generated an optimized transduction protocol for our two-vector and large payload RB-340-1F platform (Fig. 1c).

In this protocol, T cells were stimulated in anti-CD3 and anti-CD28 pre-coated plates in T cell culture medium supplemented with recombinant human IL-7 and IL-15. LdCK vector was added to the T cell culture on day 1 (18–24 h) (MOI = 10), CAR-TEV vector was added on day 2 (MOI = 20). Lentiviral vectors were washed out on day 5. Meanwhile, T cell stimulation was withdrawn via transferring cells to non-coated plates. To enrich double transduced cells, GFP and mCherry double positive cells were sorted on day 6. These double positive cells were cultured and expanded until day 14 or day 21. With this protocol, we achieved around 4%–9% double transduced cells on day 6, depending on the donors (data not shown).

### RB-340-1F lentiviral transduction of human primary T cells is promoted by BX795 treatment

To promote RB-340-1F T cell transduction efficiency, we tested different strategies, including BX795 treatment. BX795 was added to T cells along with lentiviral vectors and the other transduction enhancer Transplus. The final concentration of BX795 in cell culture is 4 µM. Six hours after transduction, BX795 concentration is diluted to 1.3 µM due to the supplement of more fresh medium to cells. For RB-340-1F, BX795 was added twice along with two lentiviral vectors on day 1 and day 2 (see “Methods”, Fig. 1c). On day 5, BX795 was washed out. No more BX795 treatment was needed after cell transduction.

**Fig. 2:**
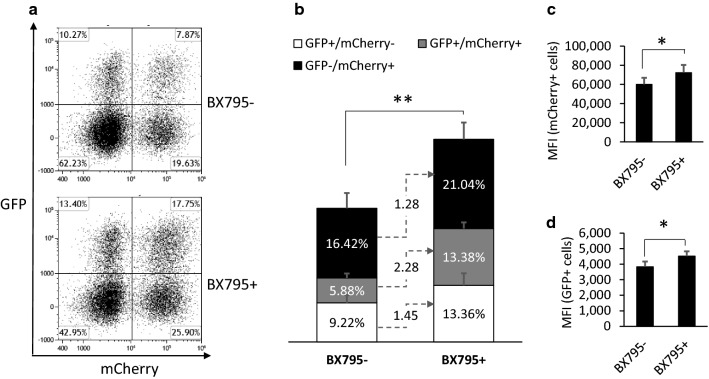
Effect of BX795 treatment on RB-340-1F lentiviral vectors transduction efficiency. **a** Transgene expression was assessed by flow cytometry on day 6. **b** T cell transduction rate with the absence of BX795 (BX795-) or presence of BX795 (BX795+). GFP+/mCherry-: GFP single positive population. GFP+/mCherry + : GFP and mCherry double positive population. GFP-/mCherry+ : mCherry single positive population. **c** MFI of mCherry in CAR-TEV-mCherry transduced cells. **d** MFI of FITC in LdCK-GFP transduced cells. All results are the averages of four donors, except for figure A, which is flow cytometry plot from a representative donor. *p < 0.05; **p < 0.01

We tested BX795 treatment with T cells from four donors. The dot plots in Fig. 2a display transgene expression assessed on day 6 by flow cytometry from one representative donor. Figure 2a shows that BX795 treatment promoted both LdCK-GFP and CAR-TEV-mCherry transduction rate dramatically. In the four donors, without BX795 treatment, LdCK-GFP transduction rate is around 12.4%–18.1%, CAR-TEV-mCherry transduction rate is around 17.3%–27.5%. With BX795 treatment, LdCK-GFP transduction rate was increased to 24.0%–31.2%, CAR-TEV-mCherry was increased to 28.9%–43.7%. The higher transduction rate by the second vector CAR-TEV-mCherry might be caused by higher MOI and shorter transgene sequence of the second vector. Collected and averaged data from four donors are shown in Fig. 2b. The total transduced T cells, including GFP +/mCherry-, GFP +/mCherry + , and GFP-/mCherry + cells, increased from 31.5% to 47.78%. We observed a 1.45-fold increase for GFP single positive population (GFP +/mCherry-), 1.28-fold increase for mCherry single positive population (GFP-/mCherry +), and 2.28-fold increase for the double positive population (GFP +/mCherry +), which represent the complete RB-340-1F product to be enriched for further expansion. Not only was there a significant increase in the percentage of transduced T cells, but median fluorescence intensity (MFI) of transduced cells was also statistically significantly increased by BX795 treatment (Fig. 2c, d). Fourteen days after cell sorting, GFP and mCherry double positive cells were analyzed by PCR for vector copy number (VCN). We found that VCN was increased by 1.38-fold with BX795 treatment, which indicates that the higher transgene expression was caused by more gene integration.

We sustainably saw transduction promotion with BX795 treatment in different experiments. The effect of BX795 was also observed when we worked with the other Refuge CAR-T platforms. These results clearly demonstrate that BX795 treatment enhances RB-340-1F and the other large payload lentiviral transduction of human primary T cells.

### BX795 treatment changes CD4+ :CD8+ compositions in RB-340-1F double transduced T cells

**Fig. 3:**
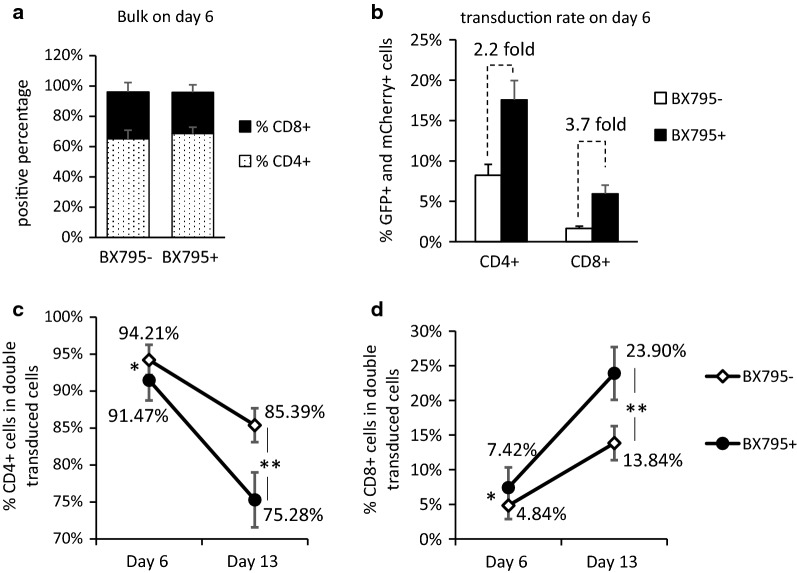
Effect of BX795 treatment on RB-340-1F transduction of CD4+ T cells and CD8+ T cells. **a** Percentage of CD4+ cells and percentage of CD8+ cells in the bulk population on day 6 before cell sorting. **b** Percentage of double transduced cells in CD4+ or CD8+ cells on day 6. BX795-: no BX795 treatment. BX795+ : with BX795 treatment. The above results are the average of four donors. **c** Percentage of CD4+ cells in double transduced cells on day 6 and day 13. **d** Percentage of CD8+ cells in double transduced cells on day 6 and day 13. BX795-: no BX795 treatment. BX795+ : with BX795 treatment. The above results are the average of three donors. *p < 0.05; **p < 0.01

To further dissect the effect of BX795 on transduced T cells, we performed T cell phenotyping analysis on day 6 before cell sorting. We found that BX795 treatment did not obviously change CD4+ :CD8+ compositions in the bulk population (the mixture of transduced and non-transduced cells) on day 6 (Fig. 3a). When gating on CD4+ cells or CD8 + cells, we saw transduction promotion in both populations. In the CD4+ population, the percentage of double transduced cells was increased from 8.22% to 17.56%, which is 2.2-fold change. In CD8 + population, the percentage of double transduced cells was increased from 1.65% to 5.94%, which is 3.7-fold (Fig. 3b). The higher fold change in CD8 + cells was consistently observed in all our experiments, and was also observed when BX795 was applied to the other Refuge CAR-T platforms (data not shown). These results indicate that BX795 might promote lentiviral transduction better in CD8+ cells, or transduced CD8+ cells grow faster with BX795 treatment. Since we did not see higher CD8+ ratio in bulk population on day 6 (Fig. 3a), we speculate that BX795 does not promote CD8 + cell growth. The mechanism underlying the different promotion extent between CD4+ cells and CD8+ cells remains not clear.

When gating on double transduced T cells, we saw higher CD8+ ratios in the BX795 treated group comparing to the group without BX795 treatment (Fig. 3d). The difference is enlarged along with T cell expansion after cell sorting. One week after cell sorting (day 13), the percentage of CD8+ cells in BX795 treated group became 23.90%, which is much higher than 13.84% of CD8+ ratio in the group without BX795 treatment (Fig. 3d). Meanwhile, the percentage of CD4 + cells in double transduced cells dropped from 85.39% to 75.28% (Fig. 3c). Although we are not sure if BX795 treatment affects lentiviral transduction of CD4+ cells and CD8+ cells in different extent or affects growth rate of transduced CD8+ cells, there is no doubt that BX795 treatment increased CD8+ ratio in RB-340-1F double transduced cells.

### BX795 treatment does not dramatically affect transduced T cell expansion and designated CAR-T cell function

**Fig. 4:**
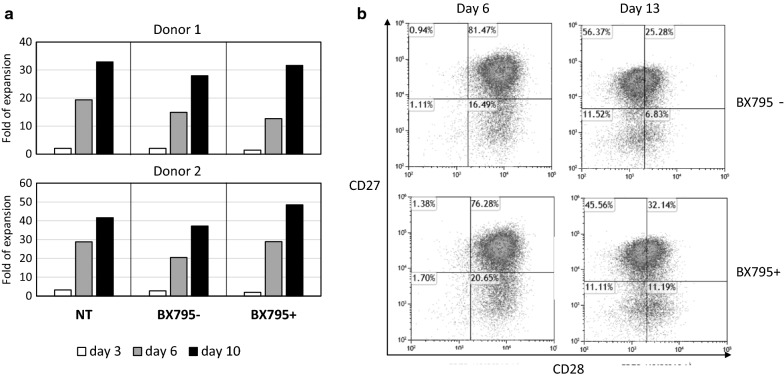
Cell growth rate and CD27/CD28 expression of transduced T cells. **a** T cell expansion rate on day 3, day 6, and day 10, with the absence or presence of BX795. RB-340-1F transduction was performed on day 1 and day 2 as described in method, including non-transduced (NT) group as a control. Cell sorting is not carried out in this experiment. Fold of expansion is calculated by dividing the actual cell number on designated day with the cell number counted on day 1. **b** Expression of CD27 and CD28 in RB-340-1F double transduced cells on day 6 and day 10. Experiments were done with more than three donors. Data presented is from one representative donor

The previous study in NK cells reported that BX795 treatment did not cause immediate toxic effects on NK cells up to 10 µM [28]. In this study, we used an even lower concentration, 4 µM BX795, in all the experiments, and did not observe obvious cell death during or after BX795 treatment. The viability of BX795-treated cells was around 90% and did not show difference compared with non-treated cells. We recorded T cell growth rate at various time points (day 3, day 6, day 10), and found that cell expansion rate in the BX795 treated group is comparable to that in the non-BX795 treated group (Fig. 4a). Cell surface markers CD27 and CD28 are frequently used to define stages of T cell differentiation and are related with T cell activation and expansion [35–37]. We detected CD27 and CD28 expression in double transduced T cells on day 6 and day 13, and did not observe obvious change of CD27/CD28 expression pattern when BX795 was used (Fig. 4b). These results indicate that using BX795 treatment does not affect T cell expansion dramatically, and may not cause earlier T cell senescence or terminal differentiation.

**Fig. 5:**
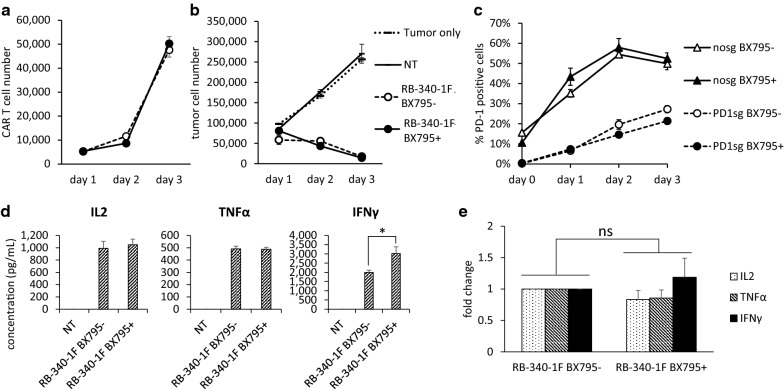
Functional assay of RB-340-1F double transduced cells. **a** Numbers of CAR + T cells existing in co-culture during 3 days killing assay against Fadu/PD-L1. **b** Numbers of Fadu/PD-L1 cells existing in co-culture during 3 days killing assay with non-transduced T cells (NT), RB-340-1F double transduced cells treated with or without BX795. **c** PD-1 positive percentage in double transduced T cells during 3 days killing assay against Fadu/PD-L1. Nosg groups are T cells transduced with LdCK-GFP vector and CAR-TEV-mCherry control vector without carrying any sgRNA sequence. PD1sg groups are RB-340-1F that contains LdCK-GFP and CAR-TEV-mCherry carrying PD-1 sgRNA sequence. **d** The concentration of secreted IL2, TNFα, and IFNγ in killing assay supernatant collected on day 1. All above results are the averages of triplicate wells. They are representative of five experiments done with T cells from two donors. **e** The average results of cytokine release from five experiments. To emphasize the fold change, cytokine concentrations in BX795 non-treated group are all normalized to 1. *p < 0.05; ns, not significant

We also investigated whether BX795 treatment presents any functional concerns regarding HER2 CAR-T cell cytotoxic capacity and target gene PD-1 knock down, which are the two essential components of RB-340-1F. Fadu, a HER2-positive oropharyngeal cancer cell line, was used for functional testing as the target cells and co-cultured with RB-340-1F double transduced T cells. To magnify PD-1/PD-L1 interactions, Fadu cells were engineered to constitutively express programmed cell death ligand-1 (Fadu/PDL-1). During 3 days killing assay analysis, we saw comparable CAR-T cell growth in BX795 non-treated and treated groups (Fig. 5a). And we did not observe any alteration in cellular cytotoxicity against Fadu/PD-L1 cells after treatment with BX795 (Fig. 5b). Moreover, target gene PD-1 knockdown was still effective. And BX795 treated group always showed a little bit better PD-L1 knock down efficiency compared with non-treated group (Fig. 5c). This subtle difference might be caused by higher PD1sgRNA or dCas9 protein expression in BX795 treated transduced cells as indicated in Fig. 2c–d.

Additionally, we detected cytokine release, including IL2, TNFα, and IFNγ, on day 1 of killing assay. Results shown in Fig. 5d are from one donor, and are the averages of triplicate wells. These results indicated that IL2 and TNFα secretion is similar among BX795 treated and non-treated groups, but IFNγ secretion is higher in RB-340-1F cells treated with BX795 (Fig. 5d). Since BX795 was withdrawn from T cell culture as early as on day 5. While T cell killing assay was performed on day 14 or on day 21. The higher secretion of IFNγ that we observed may not be directly caused by the inhibition effect of BX795. To confirm this result, we did Fadu/PD-L1 killing assay experiments five times with cells collected from two donors. The average results show that RB-340-1F cells treated with BX795 secret lower level of IL2 and TNFα, and higher level of IFNγ (Fig. 5e). However, all the differences are not significant based on t test.

The above results demonstrate that the use of BX795 to do RB-340-1F lentiviral transduction of human primary cells did not cause dramatic change in cell growth and designated cell function, including CAR-T cytotoxic activities and target gene knock down.

### Lentiviral transduction of human primary T cells with BX795 treatment is viral dose dependent

**Fig. 6:**
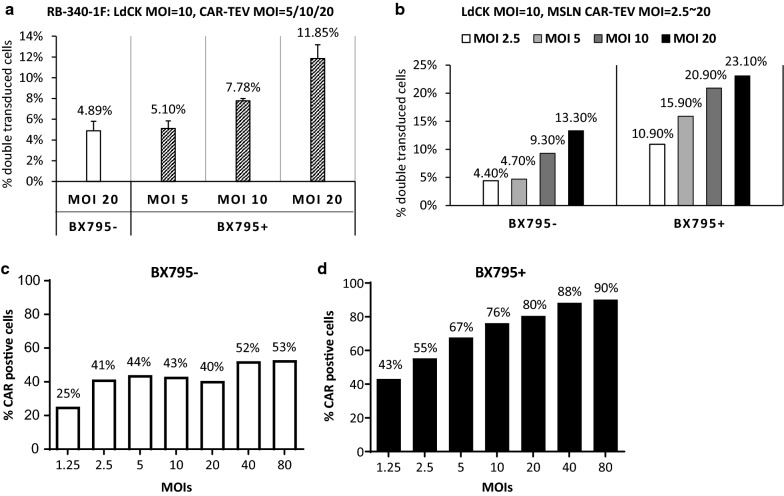
Viral dose dependent transduction effect with BX795 treatment. **a** The percentage of double transduced cells with LdCK-GFP MOI 10, and CAR-TEV-mCherry MOI 5, 10, and 20, in the absence or presence of BX795. Data are the average of four experiments done with cells from two donors. **b** The percentage of double transduced cells with LdCK MOI 10, and MSLN CAR-TEV MOI 2.5, 5, 10, and 20, in the absence or presence of BX795. Data from one representative donor. **c**, **d** Virus dose-escalation for MSLN CAR-TEV transduction in human primary T cells in the absence or presence of BX795. Data from one representative donor

To assess the relationship between virus dose and infection efficiency when BX795 is used, lentiviral vectors were added to human primary T cells with different MOIs. In the following experiments, the first virus LdCK MOI is fixed at 10, only the second virus CAR-TEV is tested with different MOIs. For RB-340-1F, we tested CAR-TEV MOI 5, 10, and 20, combined with BX795 treatment. The data show that cell transduction rate is dependent upon viral dose (Fig. 6a). The percent of double transduced cells increased with the addition of more virus. We observed the same viral dose dependent pattern when we worked with MSLN CAR/CRISPRi circuit, which shared the same LdCK vector but replaces HER2 CAR in RB-340-1 with MSLN CAR (Fig. 6b). We fixed LdCK MOI at 10, and tested MSLN CAR-TEV MOI 2.5, 5, 10, and 20, with or without BX795. It is clear that cell transduction rate is also viral dose dependent, and use of BX795 promotes transduction rate no matter what the virus dose is. Similar as RB-340-1 with no fluorescent labels (data not shown), MSLN CAR/CRISPRi circuit showed higher transduction efficiency than RB-340-1F, which is around two-fold increase (Fig. 6a, b).

In both studies, we observed that lentiviral transduction of human primary T cells with BX795 treatment is viral dose dependent. Moreover, we noticed that the double transduction rate using MOI 5 with BX795 treatment is similar as, or even higher than the transduction rate using MOI 20 without BX795 treatment (Fig. 6a, b). From manufacturing prospect, BX795 might be a good solution to reduce requirements of lentiviral vectors, thereby reduce the costs of vectors production.

Interestingly, when MSLN CAR-TEV vector was single transduced into human primary T cells without BX795 treatment, the transduction rate reached a plateau around 40% with MOIs up to 2.5 (Fig. 6c). Increasing MSLN CAR-TEV MOI did not improve transduction rate. When MOI went up as high as 80, only 53% cells were transduced. However, when BX795 was incorporated, MSLN CAR-TEV transduction rate increased significantly and showed a viral dose dependent pattern (Fig. 6d). Transduction rates up to 76% could be reached with MOI 10. This result indicates that not only multiple vectors, but also single vector transduction can benefit from use of BX795.

## Discussion

Low viral transduction efficiency is a common challenge faced by academic and industrial researchers working on T cell engineering. In this study, we focused on our product, RB-340-1F, which combines CAR-T therapy with inducible PD-1 knockdown mediated through CRISPRi. It is clear that short time BX795 treatment along with lentiviral vectors infection promoted RB-340-1F transduction of human primary T cells. For single lentiviral vector transduction, we observed that BX795 treatment promoted MSLN CAR-TEV transduction (MOI = 1.25–80), and also overcame the 40–53% transduction plateau (Fig. 6c, d). However, we have not performed a systematic study on the effect of BX795 with single and small payload lentiviral vectors. In a control experiment, we applied BX795 along with conventional HER2 CAR lentiviral vector (MOI = 0.2–0.5) to human primary T cells, and we did not see any enhancement effect with BX795 treatment (data not shown). In contrast to this experiment, when we applied BX795 with a small size Luc-EGFP lentiviral vector (MOI > 1), transduction efficiency was increased around two-folds. We speculate that the reason we did not see transduction promotion for conventional HER CAR is because the vector MOI is too low. We believe that BX795 treatment should have much broader use, not only limited to Refuge CAR/CRISPRi circuits products.

In this study, BX795 stayed in T cell culture for 4 days at a concentration of 4 µM for 12 h, and the concentration of 1.3 µM in the rest of the 4 days. Except for lentiviral transduction efficiency, other changes may be caused by BX795 in the treated cells. Among T cell characters or parameters that could be easily measured, no change was observed. For instance, BX795-treated T cells kept high viability and similar growth rate as T cells without BX795 treatment. T cell phenotyping before cell sorting on day 6 showed that there was no obvious change on the expression of CD4, CD8, CD27, and CD28 in the bulk population. The sorted double transduced RB-340-1F cells showed comparable CAR-T cytotoxic activity and target gene knock down as RB-340-1F cells without BX795 treatment. However, some differential effects on T cells were observed. For instance, BX795 treatment increased CD8+ ratio in RB-340-1F double transduced cells. More studies need to be done to uncover the mechanisms underlying this effect.

BX795 was originally developed as a small molecule inhibitor of 3-phosphoinositide-dependent protein kinase 1 (PDK1) [38]. It was subsequently found to specifically block the TBK1 and IKKɛ complex [27]. It is well known that TBK1 and IKKɛ regulate the production of type 1 IFNs during viral infection [22, 23]. Moreover, type 1 IFNs have been reported to influence the fate of CD4+ and CD8+ T cells during the initial phase of antigen recognition, and are also required for CD8+ T cell expansion and memory formation during lymphocytic choriomeningitis (LCMV) infection contributing to the formation of CD8+ T cell memory and effector function in response to vesicular stomatitis virus infection [39–41]. Although the effect of type 1 IFNs on T cells in the above situations may not be exactly the same as in conditions related to lentiviral transduction of human primary T cells, it should be recognized that blocking TBK1 and IKKɛ signaling via BX795 may alter T cell phenotype and function. Further characterization of signaling pathways involved in this response is needed to incorporate BX795 into T cell product manufacturing.

## Conclusions

This study demonstrates that BX795 treatment promotes lentiviral transduction of combinatorial CAR/CRISPRi circuit RB-340-1F into human primary T cell, with the averages at 2.3-fold change. After BX795 treatment, the percentage of CD8+ cells in RB-340-1F double transduced T cells was increased, while cell growth and CAR-T cell function were not dramatically affected. This observation indicates that the use of BX795 is an effective and safe approach to promote RB-340-1F T cell production. Moreover, single vector transduction can also benefit from using of BX795 by overcoming the transduction efficiency plateau often observed in human primary T cells. This approach might have broader use for other T cell or other immune cell therapy products.

## Data Availability

All data are available in the manuscript or upon request to the authors.

## Abbreviations

TBK1: TANK-binding kinase 1
IKKɛ: IƙB kinase ɛ
IFNs: Interferons
NK cells: Natural killer cells
CAR: Chimeric antigen receptor
CRISPR: Clustered regularly interspaced short palindromic repeats
dCas9: Nuclease-deactivated Cas9
ΔU3: Deleted U3 region
EF1α: Human elongation factor-1 alpha promoter
GFP: Green fluorescent protein
HER2: Human epidermal growth factor receptor 2
Krab: Kruppel-associated box
LAT: Linker for activation of T cells
LTR: Long terminal repeat
mU6: Mouse U6 promoter
NES: Nuclear export sequence
PD-1: Programmed cell death protein 1
PD-L1: Programmed death ligand-1
scFv: Single chain variable fragment
sgRNA: Short guide RNA
TCS: TEV cutting site
TEV: Tobacco etch virus
TSS: Transcription start site
WPRE: Woodchuck hepatitis virus posttranscriptional regulatory element
LDLR: Low-density lipoprotein-receptor
MFI: Median fluorescence intensity
E:T: Effector-to-target ratio
VCN: Vector copy number
